# Driver Assistance System for Passive Multi-Trailer Vehicles with Haptic Steering Limitations on the Leading Unit

**DOI:** 10.3390/s130404485

**Published:** 2013-04-03

**Authors:** Jesús Morales, Anthony Mandow, Jorge L. Martínez, Antonio J. Reina, Alfonso García-Cerezo

**Affiliations:** Dpto. Ingeniería de Sistemas y Automática, Universidad de Málaga, 29071 Málaga, Spain; E-Mails: jesus.morales@uma.es (J.M.); amandow@uma.es (A.M.); ajreina@uma.es (A.J.R.); ajgarcia@uma.es (A.G.-C.)

**Keywords:** driver assistance system, articulated vehicles, curvature limitations, virtual tractor, trailer, drive-by-wire, jackknife avoidance

## Abstract

Driving vehicles with one or more passive trailers has difficulties in both forward and backward motion due to inter-unit collisions, jackknife, and lack of visibility. Consequently, advanced driver assistance systems (ADAS) for multi-trailer combinations can be beneficial to accident avoidance as well as to driver comfort. The ADAS proposed in this paper aims to prevent unsafe steering commands by means of a haptic handwheel. Furthermore, when driving in reverse, the steering-wheel and pedals can be used as if the vehicle was driven from the back of the last trailer with visual aid from a rear-view camera. This solution, which can be implemented in drive-by-wire vehicles with hitch angle sensors, profits from two methods previously developed by the authors: safe steering by applying a curvature limitation to the leading unit, and a virtual tractor concept for backward motion that includes the complex case of set-point propagation through on-axle hitches. The paper addresses system requirements and provides implementation details to tele-operate two different off- and on-axle combinations of a tracked mobile robot pulling and pushing two dissimilar trailers.

## 1. Introduction

Advanced driver assistance systems (ADAS) are gaining more and more attention as a key technology to increase driver comfort and safety. This is a wide research area that includes adaptive cruise control [1], navigation [2], and perception of vehicles [3], pedestrians [4] or traffic signs [5]. A common feature of ADAS is using cameras [6,7] and other sensors [8] to improve driver awareness. Furthermore, drive-by-wire systems [9] allow to implement haptic human/machine interfaces like actuated steering wheels [10,11]. Vehicles with one or more trailers, such as trucks or multi-body combinations for goods and passengers, can also benefit from ADAS because their maneuvering is complex even for skilled drivers [12,13].

**Figure 1.**
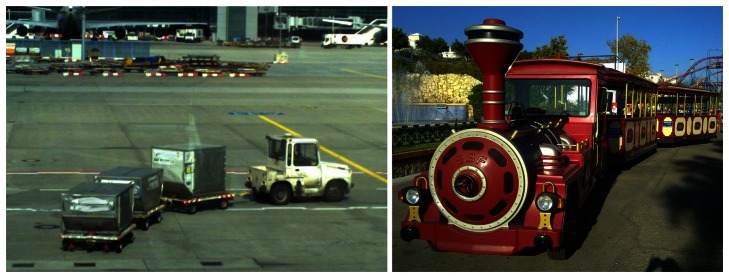
Examples of multi-trailer systems: Baggage carriers in an airport (**left**) and a tourist road train (**right**).

The position of hitches is relevant when pushing or pulling trailers [14]: A trailer hitch is “on-axle” if it lies on the preceding unit's rear axle, and is “off-axle” otherwise. For example, most caravans have a passive off-axle hitch. Furthermore, combinations of passive on- and off-axle trailers are frequent in vehicles such as airport luggage carriers and tourist road trains, whose wagons are usually made up of a front off-axle trailer and a rear on-axle trailer (see Figure 1).

In forward motion, the driver has to steer carefully in order to avoid inter-unit collisions [15]. Backwards, jackknife avoidance is a benchmark nonlinear control problem that has been approached with feedback linearization [16], fuzzy control [17,18], or switching control [19]. However, many of these theoretical approaches are difficult to implement and to tune [20,21] so practical solutions are necessary [22,23]. In this sense, driver assistance is a significant practical application [12], especially because unaided reverse driving with multiple passive trailers becomes utterly difficult, if not impossible.

Haptic handwheels are an effective interface for steering assistance [10]. Thus, motorized steering-wheels have been employed as a warning mechanism for lane departure [24] and road obstacles [25]. Furthermore, force feedback can improve the driver steering performance, which has been applied for lane-keeping [26], adapting to dangerous road conditions [27], and backward parking [28].

In addition to jackknife, drivers of articulated vehicles have difficulties in surveying the rear part of the vehicle, which not only adds to the complexity of backward maneuvering but also endangers pedestrians and other road users. Vehicle mounted camera systems offer a solution for blind spot monitoring [29] and parking assistance [6,7]. Precisely, rear-view cameras have been employed to enhance driver perception in a truck and trailer [30,31].

In spite of these steering and perception difficulties, not many works have focused on ADAS for articulated and multi-articulated vehicles. Feedback and feedforward control for a steered trailer can help the driver to reduce off-tracking in long trucks [13]. For passive trailers, a neural network predictor has been proposed to assist the driver in anticipating jackknife situations [32]. Furthermore, the ADAS proposed in [12] combines motion control with a driver interface to push homogeneous off-axle passive trailers with a reversed car. Recently, we proposed an ADAS system for backward maneuvers with off-axle trailers [33] that integrated the curvature limitations and virtual tractor concepts [34]. A further theoretical development has extended virtual tractor steering by addressing the difficulty of propagating set-points through on-axle hitches, which cannot be achieved directly [35,36].

The major contribution of this paper is to complete [33] by incorporating [36] into a comprehensive drive-by-wire ADAS solution that is useful for reverse and forward maneuvers with combinations of on- and off-axle trailers. Unsafe steering commands are prevented by conveying curvature limitations to the driver through a haptic steering wheel. In reverse, the handwheel and pedals can be used as if the vehicle was driven from the back of the last trailer, *i.e.*, a virtual tractor, with visual aid from a rear-view camera. This new ADAS has been implemented to tele-operate two different off- and on-axle combinations of a tracked mobile robot pulling and pushing a pair of dissimilar trailers.

The paper is organized as follows. Section 2 discusses the requirements for a multi-trailer ADAS. Section 3 describes the case study for a two-trailer robotic vehicle where the ADAS has been implemented, and discusses experimental results. Finally, Section 4 presents conclusions and future work.

## 2. Driver Assistance System Requirements

This section discusses sensors and other hardware requirements for the multi-trailer ADAS. From the driver's standpoint, the ADAS specifications are the following:

- It should allow forward and reverse driving with combinations of on- and off-axle trailers without the driver minding inter-unit collision or jackknife.
- The driver should be aware of curvature limitations through the steering wheel.
- Driving in reverse can be done as if it was forward driving from the last trailer.

**Figure 2.**
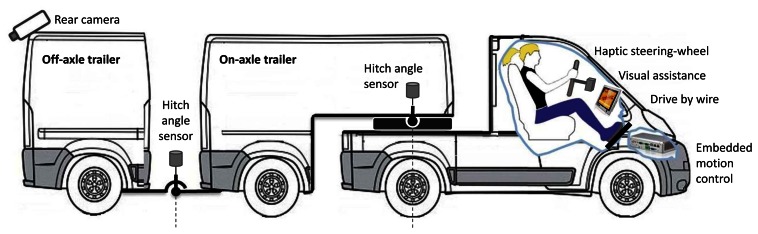
Overview of the multi-trailer ADAS concept.

These specifications relate to all the driver assistance categories [28]: Cognitive assistance to enhance driver perception; mobility assistance, associated with vehicle motion control, and operation assistance to support adequate operation of the driver. The proposed ADAS solution, outlined in Figure 2, involves different technological requirements for each of these categories, as discussed below.

### 2.1. Cognitive Assistance

Driving in reverse from the perspective of the last trailer demands visual assistance. To this end, a video camera has to be placed on the back of the last trailer to display video images on a dashboard screen (see Figure 2). A wireless camera would allow a simple and flexible set-up in case of changes in the multi-trailer configuration [31]. Furthermore, pan-tilt-zoom (PTZ) cameras can allow adjustment from the driver's display. Mounting the camera at an overhead position can improve the perspective of the area behind the articulated vehicle and part of the last trailer can be included in the image as a reference.

During forward motion, the display can be deactivated to avoid distracting the driver's attention [37]. This deactivation does not apply for tele-operated vehicles, where a forward pointing camera is also required.

### 2.2. Mobility Assistance

Backward motion control of an articulated vehicle can be implemented by considering the last trailer as a virtual tractor with non-holonomic constraints that moves forward [34,36]. With visual feedback, the vehicle can be steered backwards as if the driver was sitting in the last trailer.

To achieve this, drive-by-wire controls (*i.e.*, pedals, reverse/forward selector, and steering wheel) are necessary. Moreover, an embedded processor is required to interface with the driver controls and to transform motion commands in real time (see Figure 2).

Transforming virtual linear speed and curvature set-points into control inputs for the actual vehicle entails a kinematic propagation starting from the last trailer. Propagation through on-axle trailers has a transient introduced by a proportional control law that is not necessary for off-axle trailers [36]. In any case, propagation depends on the current values of the hitch angles. Therefore, it is necessary to measure these angles continuously with appropriate sensors (e.g., encoders or potentiometers) and to transmit them to the embedded controller.

### 2.3. Operation Assistance

Imposing constant curvature limitations for single-axle multi-trailer systems was first proposed to prevent inter-unit collisions in forward motion [15]. This was extended to obtain curvature limitations for a virtual tractor to avoid jackknife in backward motion [34]. In both cases, the curvature limitations are computed off-line for a given configuration of the articulated vehicle.

The procedure to obtain curvature limitations ±*γ_m_* for the leading unit comprises two stages. First, the kinematic parameters and the mechanical limits of the hitches are employed for a steady-state study that recursively bounds curvature starting from the last unit. Then, this result is refined by a simulated transient analysis that accounts for vehicle actuator dynamics and non-minimum phase responses of multi-trailer systems.

The curvature limitation method presented in [15,34] considers differential drive steering of the tractor, where zero turning radius is possible. As most commercial vehicles use Ackermann steering, which has a mechanical curvature bound, applicability of the method has to be properly adapted. In the forward case, the most restrictive limitation between the computation proposed in [15] and the mechanical bound is chosen. In backward motion, the procedure to compute virtual tractor curvature limitations [34] can be generalized to Ackermann steering by using the mechanical tractor limit to initialize the steady-state study.

Curvature limitations can be incorporated into the ADAS by using a haptic steering wheel with force feedback so that the driver feels these limits as if they were mechanical bounds (see Figure 2). Different limitations are scheduled by the embedded processor depending on the actual configuration of the articulated vehicle and on its motion direction.

To implement the curvature limitation ±γ*_m_*, a bound ±*θ_m_* has to be defined in the haptic steering-wheel angle *θ*. In this way, the curvature set-point γ*_s_* for the leading unit is computed from *θ* as:

(1) $$\gamma_{s}=\theta \frac{\gamma_{m}}{\theta_{m}}$$

Furthermore, force feedback can inform the driver about the proximity to the handwheel limits. Thus, the torque *τ* applied by the driver within ±*θ_m_* can be specified as a centering spring-damper effect:

(2) τ=Jθ″+bθ′+kθ

where *J* is the moment of inertia of the handwheel, *b* is the dynamic friction coefficient, and *k* is the stiffness constant.

## 3. Case Study

### 3.1. Auriga-α and Two Trailers

**Figure 3.**
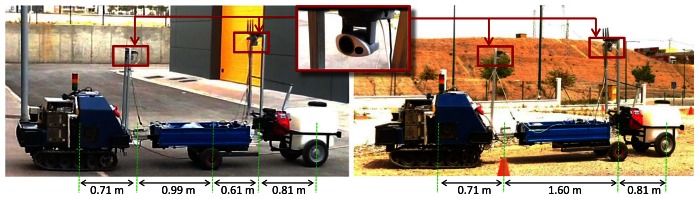
Auriga-α two-trailer vehicle with off/off-axle (**left**) and off/on-axle (**right**) setups with kinematic parameters and location of front and rear IP cameras. Inset: camera detail.

The proposed ADAS system has been tested to tele-operate the tracked Auriga-α mobile robot with two single-axle passive trailers: a load carrier and a sprayer (see Figure 3). The second trailer can be either on-axle or off-axle by manually repositioning the first trailer's axle. This way, two different configurations have been tested: *off*/*off*, where both joints are off-axle, and *off*/*on*, where the second is on-axle. The kinematic parameters are shown in Figure 3, where the null distance between the axle of the first trailer and its rear hitch is not shown in the *off*/*on* configuration.

Auriga-α weights 258 kg and its dimensions are 1.24 m (length), 0.75 m (width) and 0.84 m (height). Two geared DC motors with incremental shaft encoders provide skid-steer locomotion. An approximated differential drive kinematic model [38] has been obtained for odometric estimations and control. The maximum speed of the vehicle (1 m/s) can only be achieved in straight-line motion. An on-board digital signal processor (DSP) controls motor speeds every 10 ms and gives odometric data every 30 ms.

Hitch angles are measured through inter-unit draw-wire displacement sensors. The mechanical inter-unit collision limits of the hitches are *θ*_1_*_m_* = ±68° and *θ*_2_*_m_* = ±43.6° for the first and the second trailer, respectively. Table 1 shows the curvature limitations *γ_m_* computed for the leading unit with each trailer configuration and motion direction. Details about the obtention of these constants can be found in [15] for forward motion, and in [34] for reverse.

In each experiment, the origin of the global frame is initialized at the starting position of Auriga-α. Then, the pose of the tractor is recorded every 270 ms by correcting odometric estimations with an accurate laser scan matching technique [39]. To this end, an onboard Sick LMS 200 rangefinder is installed in the front end of the vehicle (see Figure 3). Moreover, trailer poses can be kinematically deduced from the mobile robot position and hitch angle measurements. This recorded information is not available for the driver as it is not part of the ADAS.

### 3.2. Remote ADAS Implementation

Auriga-α does not allow on-board driving, so a remote ADAS interface is managed by a dedicated PC with a wireless connection. Thus, visual assistance is required for both forward and reverse driving.

**Figure 4.**
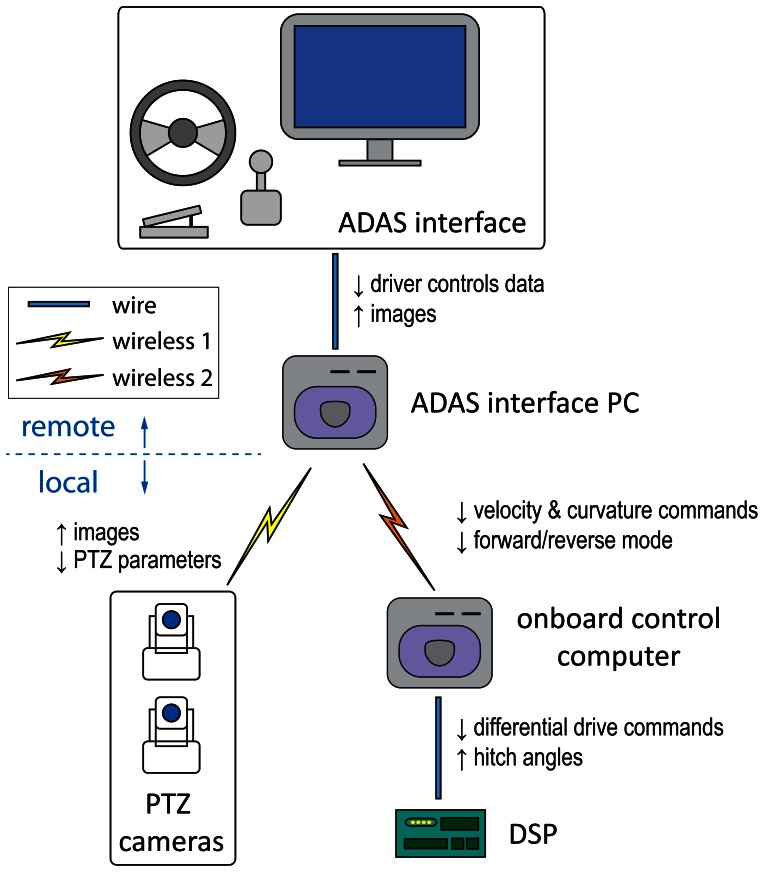
Drive-by-wire hardware architecture and information flow.

The implemented drive-by-wire hardware architecture is summarized in Figure 4. An onboard control processor with a real-time operating system issues differential drive commands for the DSP motor controller and receives hitch angles. This processor implements the virtual tractor set-point transformation for backward motion. Equations for propagation through off-axle and on-axle hitches can be found in [36].

**Figure 5.**
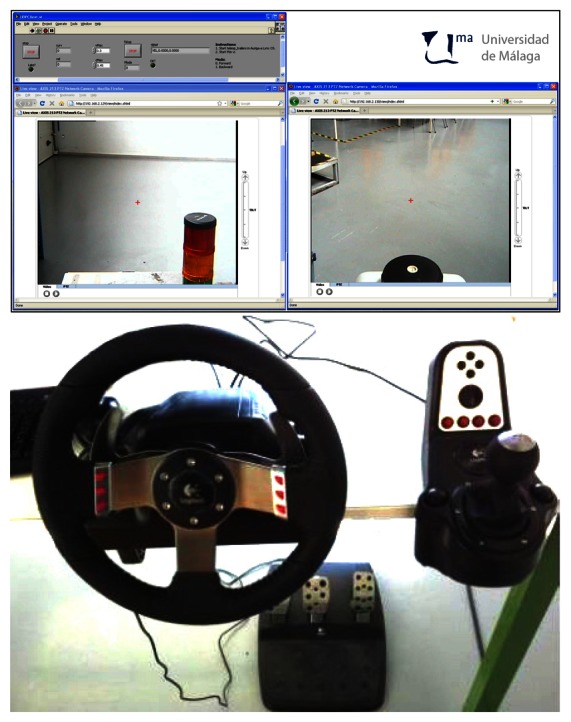
ADAS Driver Interface: Dashboard display screen capture (**top**), and drive-by-wire controls (**bottom**).

The ADAS interface is shown in Figure 5. It consists of a display for camera images and a commercial kit of driver controls: a haptic steering wheel, a manual lever, and pedals. Values from the driver controls are read with a LabVIEW application running in the interface PC.

The display offers images from two PTZ cameras for visual feedback: one mounted on the last trailer and another on Auriga-α (see Figure 3). These images are received through a local wireless TCP-IP network, which is independent from the link between the PCs.

The manual lever has been programmed to select the forward and reverse driving modes. As for pedals, only the accelerator is used to produce linear speed set-points for the leading unit *υ_s_*. The steering wheel has an optical encoder and motorized force feedback. The steering wheel rotation limit has been established as *θ_m_* = 60°. Then, the curvature set-point for the leading unit is computed using Equation (1) with the appropriate value of γ*_m_* in Table 1. The moment of inertia of the handwheel has been identified experimentally as *J* = 2.50 · 10^−4^ kgm^2^. The rest of haptic parameters in Equation (2) have been set to *b* = 7.18 · 10^−3^ kgm^2^/s, and *k* = 7.12 · 10^−2^ kgm^2^/s^2^.

### 3.3. Experimental Results

**Figure 6.**
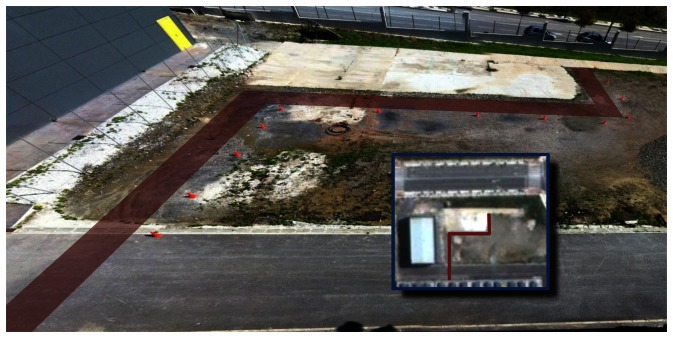
View of the experimental site where the lane has been highlighted in red. Inset: Satellite view.

**Figure 7.**
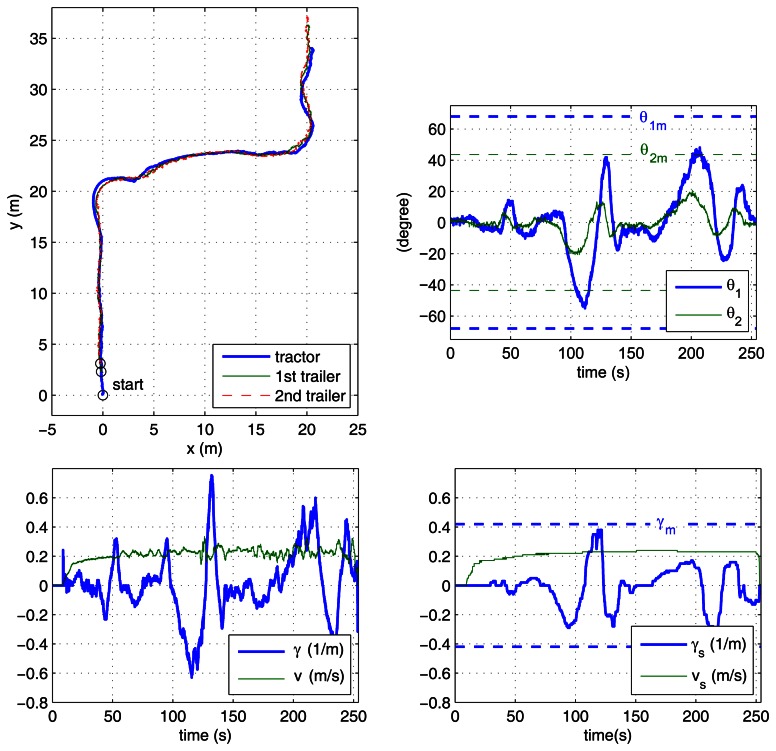
Reverse driving with the off/on axle setup: paths of the tractor and trailers (**top left**); hitch angles with corresponding mechanical limits (**top right**); driver commands and curvature limitations (**bottom right**); speed and curvature of the actual tractor (**bottom left**).

**Figure 8.**
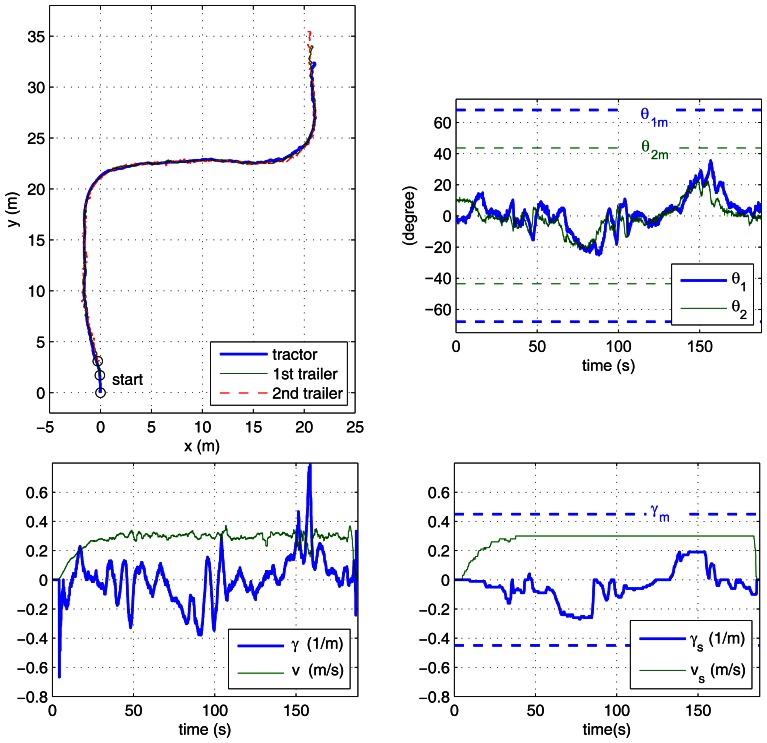
Reverse driving with the off/off axle setup: paths of the tractor and trailers (**top left**); hitch angles with corresponding mechanical limits (**top right**); driver commands and curvature limitations (**bottom right**); speed and curvature of the actual tractor (**bottom left**).

**Figure 9.**
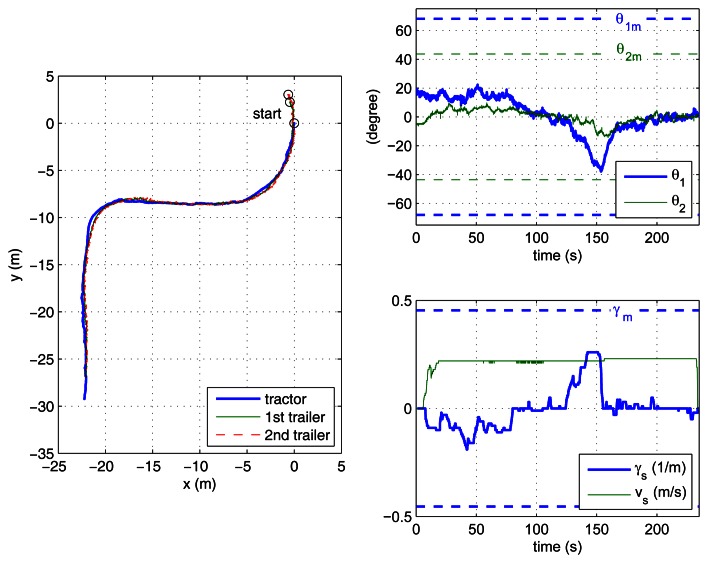
Forward driving with the off/on axle setup: paths of the tractor and trailers (**left**); hitch angles with corresponding mechanical limits (**top right**); driver commands and curvature limitations (**bottom right**).

**Figure 10.**
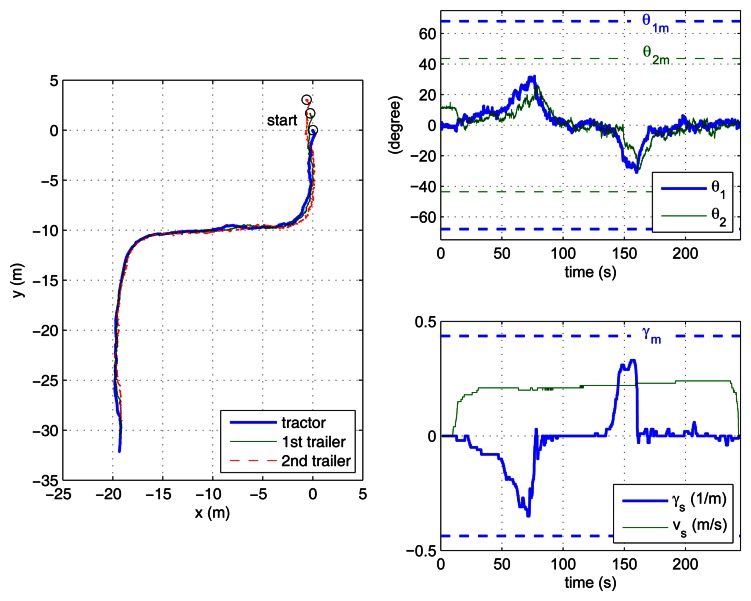
Forward driving with the off/off axle setup: paths of the tractor and trailers (**left**); hitch angles with corresponding mechanical limits (**top right**); driver commands and curvature limitations (**bottom right**).

In the case study experiments, the driver visually follows a lane delimited by red cones with an approximate width of 3 m and about 60 m long, which includes two 90° turns. This lane has been overprinted on the photographs of the outdoor experiment site shown in Figure 6. For reverse motion, the same lane is followed in the opposite direction. Two different zones have to be traversed: a smooth paved road and an irregular soil terrain. The presented results correspond to the driver using the remote ADAS to tele-operate the vehicle with both two-trailer setups. With each setup, the driver first steered backwards (Figures 7 and 8) and then returned with forward motion (Figures 9 and 10). The corresponding videos are available as supplementary files.

Figures 7 to 10 show the actual paths recorded for Auriga-α and the trailers as well as the evolution of hitch angles, which are kept within their mechanical limits. The driver commands for the leading unit (either the actual or the virtual tractor) are shown as well. In all cases, the driver succeeded in keeping the vehicle inside the lane without inter-unit collision or jackknife in spite of the perturbations introduced by irregular terrain.

The success of the proposed ADAS is noticeable especially in the backwards experiments (Figures 7 and 8), as all our previous efforts for unaided backward driving by directly steering the actual tractor had always resulted in jackknife. In forward motion, the lane can be followed by an unaided driver by carefully steering. In this case, the ADAS contributes to driver comfort by eliminating the risk of unsafe steering commands.

The experiment where the driver had more difficulty was with the reverse off/on case (see Figure 7), which is in clear contrast with the reverse off/off case (see Figure 8). In fact, this is the only experiment where the driver tried to surpass the safe steering wheel limit *γ_m_*, around 120 s during the first turn. This difficulty can be attributed to two major causes: first, stricter curvature limitations for reverse off/on (as presented in Table 1); second, the transient due to propagation of driver commands through the on axle hitch.

Figures 7 and 8 show the speed and curvature of the actual tractor (*υ* and γ, respectively), which is acting as the last unit. It can be seen that γ never exceeds 0.8 m^−1^. This means that similar results could have been obtained using an Ackermann steering tractor with a maximum turning radius of 1.25 m.

## Conclusions

This paper has proposed an advanced driver assistance systems (ADAS) to avoid inter-unit collisions and jackknife in vehicles that pull or push one or more passive trailers. For this purpose, the ADAS incorporates safe steering bounds that are computed off-line for a particular trailer configuration and motion direction. Furthermore, reverse driving is achieved using the virtual tractor concept for combinations of off- and on-axle trailers. The proposed contribution is especially relevant for reverse driving with multiple trailers, which can become utterly difficult, if not impossible, to unaided drivers due to inherent instability. In forward motion, the ADAS simply contributes to driver comfort by not having to mind about unsafe steering commands.

The ADAS hardware consists of a rear-view camera in the last trailer to feed images to a dashboard screen, drive-by-wire controls with a feedback-force steering wheel, hitch angle sensors, and an embedded processor for motion control and virtual tractor command propagation. This solution provides all three categories of driver assistance [28]: cognitive, through visual information for reverse driving; *mobility*, by transforming virtual tractor commands; and *operation*, by avoiding unsafe steering commands through a haptic feedback.

The critical component of the haptic feedback is the steering wheel limit that avoids that the driver surpasses unsafe curvature commands. Secondarily, we have profited from the haptic properties of the handwheel to define a spring effect that simulates self-centering action and also informs about the proximity of curvature limits.

From a practical viewpoint, the proposed ADAS serves to avoid inter-unit collisions and jackknife, but it cannot guarantee maneuverability if a given multi-trailer combination demands severe curvature limitations. Furthermore, the proposed ADAS does not aim at reducing trailer off-tracking, which can be minimized with appropriate kinematic parameters [12].

The paper has offered a case study where the system has been implemented as a remote driver interface to tele-operate a two-trailer robotic vehicle. Videos of these experiments have been provided as supplementary files. In the experiments, the driver achieves a good vehicle behavior for both forward and backward driving in spite of challenging terrain perturbations. Experiments have also shown that a simple change in the position of one trailer axle has a relevant impact on backward driver maneuverability if it becomes an on-axle hitch. This difficulty is caused because propagation of set-points from a virtual tractor through on-axle joints is subject to kinematic restrictions [35,36].

Precisely, dealing with these on-axle transients to improve driver performance will be a matter for future work. Furthermore, extensive user tests would be necessary to assess the acceptance of the ADAS and could be useful to improve interface design and haptic response parameters.

## Supplementary Material



## Figures and Tables

**Table 1.** Curvature limitation *γ_m_* for the leading unit depending on motion direction and trailer configuration.

	**forward**	**backward**
off/off axle	0.44 m^−1^	0.45 m^−1^
off/on axle	0.46 m^−1^	0.40 m^−1^
